# Metagenomic Next-Generation Sequencing for Periprosthetic Joint Infections

**DOI:** 10.7759/cureus.38726

**Published:** 2023-05-08

**Authors:** Bogdan Cretu, Sergiu Iordache, Adrian Cursaru, Bogdan Serban, Mihai Costache, Catalin Cirstoiu, Razvan Spiridonica

**Affiliations:** 1 Orthopedics and Traumatology, University Emergency Hospital, Bucharest, ROU

**Keywords:** total knee, targeted antibiotics, metagenomics ngs, next-generation sequencing, periprosthetic joint infections

## Abstract

Periprosthetic joint infection (PJI) after arthroplasty is a major complication, which requires significant resources, resulting in high costs for the medical system. In recent years, significant progress has been made in the diagnosis and treatment of periprosthetic infections, the identification of the pathogen being the central element in the establishment of targeted antibiotic therapy. Next-generation sequencing (NGS) or metagenomic NGS (mNGS) represents a promising, fast alternative, with increased specificity and sensitivity compared to identification methods using conventional culture media, thus enabling an increased rate of identification of pathogenic microorganisms and antibiotic resistance genes (ARG). The purpose of this article was to highlight new molecular diagnostic methods for periprosthetic joint infections and their involvement in treatment efficiency. NGS technologies are cutting-edge techniques that may challenge the PJI diagnostic model.

## Introduction and background

Periprosthetic joint infection is a serious complication after joint replacement surgery; it requires long periods of hospitalization and re-interventions, and despite the progress recorded in terms of the diagnosis and management of periprosthetic joint infection (PJI), it still represents a challenge for medical staff. Therefore, it is an important public health problem, with significant costs. The risk of periprosthetic infection is more common in the case of knee arthroplasties, its rate varies in most centers, from 0.5% to 2% for knee arthroplasties, 0.5% to 1% for hip arthroplasties and <1% for the shoulder, the higher numbers being attributed to the increased mobility of the joint and adjacent anatomical structures [1]. Recently, a study conducted in Taiwan revealed a decrease in the infection rate in knee arthroplasties from 1.9% to 0.76% [2]. Similarly, the incidence of PJI decreased from 1.4% to 0.6% between 2008 and 2016 in a US study of 11,800 patients with hip or knee arthroplasty [3].

Next-generation sequencing (NGS) is a method of parallel sequencing of deoxyribonucleic acid (DNA) found in a sample from the host or microorganisms; the length of each reading can vary between 75 and ~10,000 base pairs, these long reads being ideal for constructing the genome, thus allowing the identification of microorganisms. The cost of this technique has decreased recently, enabling its use to become more routine [4].

The technique may be particularly useful when there is strong clinical suspicion of PJI and cultures or when other diagnostic tests are negative, which is an extremely frequent scenario in PJIs. Metagenomic next-generation sequencing (mNGS) detects the entire DNA or RNA sequence from a sample, an aspect that allows the determination of a pathogen of any type (fungi, parasites, bacteria, viruses) in 24-48 h from the sampling of the specimen, compared to the three to seven days required for the detection of bacteria in the synovial fluid [5].

The purpose of this study was to highlight the impact of periprosthetic joint infections in the healthcare system and the need to focus on the new molecular diagnostic methods, and their large-scale implementation, for the improvement of treatment efficiency and the reduction of costs.

## Review

Diagnosis

Approximately two-thirds of periprosthetic joint infection cases are due to the intraoperative inoculation of microbial agents and, depending on their virulence, they can manifest early (within four weeks after the inoculation of microorganisms) or late (between three months and three years). Early infections are manifested by the presence of local and systemic inflammatory signs and are most frequently caused by pathogens with increased virulence (*Staphylococcus aureus*, Streptococcus spp., Enterococcus spp.). As for late infections, the signs are more diminished, with pain and joint weakness, and are caused by pathogenic microorganisms with low virulence (coagulase-negative staphylococci or Cutibacterium species) [6].

In 2011, the Musculoskeletal Infection Society (MSIS) and the Infectious Diseases Society developed diagnostic criteria for periprosthetic infections [7], which were revised in 2018, with a sensitivity of 97.7% and a specificity of 99.5% [8]. The major diagnostic criteria in PJI must include either two positive cultures or the presence of a continuity solution within the visualization of the joint or the prosthesis. Minor criteria include - elevated serum levels of CRP (>1 mg/dL) or D-dimers (>860 ng/mL), ESR >30 mm/h, elevated levels of leukocytes in the synovial fluid (>3000 cells/µL), alpha defensin (cutoff >1), percentage of polymorphonuclear cells >80%, synovial CRP >6.9 mg/L and leukocyte esterase (++). Patients with a total score ≥6 are considered to have periprosthetic joint infection, and a score between 2 and 5 requires the inclusion of intraoperative criteria to confirm or refute the diagnosis. Intraoperative criteria include positive histology, presence of purulent fluid, and a single positive culture. Together with the intraoperative criteria, a total score ≥6 confirms infection, a score between 4 and 5 is considered inconclusive, and a score ≤3 refutes infection [8]. The clinical manifestations of PJI depend on the route of transmission of the infection, the nature of the infected tissue, the virulence of the pathogen, and the duration of the disease. Acute manifestations occur <3 months after the surgical intervention, being frequently caused by the direct inoculation of the microorganism intraoperatively or colonization through the postoperative wound; patients can present with severe pain, high fever, localized fever, sometimes wound effusion, and elevated CRP (pathogenic agents, in this case, being* Staphylococcus aureus *and Gram-negative bacteria). Chronic infection presents with progressive pain, skin fistula formation, and/or drainage of purulent secretions, and occurs >3 months after the intervention of arthroplasty (dissemination of the infection can be hematogenous, with a primary starting point, or by direct inoculation in the case of infectious agents with low virulence, especially in the case of coagulase-negative staphylococci, *Cutibacterium acnes*, and Corynebacterium). In order to diagnose PJI, the histology of the synovial tissue, the number of leukocytes in the synovial fluid [4], determination of the levels of systemic inflammatory markers (such as ESR, CRP, and IL-6 [9]), imaging investigations (such as standard radiographs), technetium-labeled bone scans, MRI, CT, and PET/CT have been used [10]. Recently, new techniques have been developed to identify the pathogen, the conventional methods used in the past were accompanied by a high failure rate (due to the lack of isolation of a pathogenic microorganism through the premature administration of antibiotics and seeding techniques on culture media) [11]. Recently, for the optimal diagnosis and treatment of PJI, the molecular sequencing techniques NGS and mNGS have been implemented; these can identify the infecting microbial DNA, with an increased success rate, and have a high sensitivity and specificity compared to identification on conventional culture media, which is important because such diagnostic results influence antibiotic and surgical treatment [12].

Culture-negative periprosthetic joint infection

Routine microbial culture is the standard for microorganism identification, but culture sensitivity in the context of identifying periprosthetic infections (PII) remains low, at 39-70% [13]. Failure to isolate the pathogen reduces the chance of successful treatment of culture-negative periprosthetic joint infections (CN-PJI) to less than 70%, putting patients at risk of complications [13]. The incidence of CN-PJI has been reported to range from 7% to 15%, although incidences as high as 42% have also been reported [14,15].

In the case of periprosthetic joint infections with increased resistance to antibiotic therapy, or with negative cultures, the surgical treatment is similar to that in the case of obstinate PJI, to which is added meticulous interdisciplinary collaboration with a specialist in infectious diseases. Systemic antibiotic therapy is continued until the clinical and serological signs disappear [16]. In the case of infections with a negative culture, the key to optimal diagnosis is the combination of the usual diagnostic methods with new technologies to identify the pathogenic microorganism and use targeted antibiotic therapy. In recent studies, in cases of infections where the pathogen could not be identified by means of conventional culture techniques, molecular techniques revealed an 89% isolation rate for microorganisms [17]. Six studies showed an NGS pathogen detection rate of >50% for patients with negative cultures prior to sequencing, and three studies showed a detection rate of <50% (between 9% and 31%) [4].

NGS method of diagnosis

Recently, next-generation sequencing (NGS) technologies have been applied to microbiological assays, enabling high-throughput sequencing of billions of nucleic acid fragments, performed mainly in metagenomics (MG) and metatranscriptomics (MT) with high sensitivity and specificity (Table 1). In particular, MT analysis produces gene expression data that can reveal active signaling pathways. This is superior to MG, which does not distinguish between active and inactive genes in the sample. Because MG generates sequence data from all the DNA present in the sample, this method is prone to false positives when detecting pathogens [18,19]. These false positive results occur because DNA degrades over time, making inactive microbial populations or empty MG-containing DNA detectable. The higher MT discrimination is likely to be due to the generation of sequenced data from active transcripts, avoiding potentially dead or inactive microbial populations [19]. Metagenomic NGS is a sequencing technique that has a higher yield than NGS, being able to sequence not only the complete bacterial genome but also the antimicrobial resistance genes (ARG) [20]. The newest technique is the metatranscriptomic one (MT-NGS), which, unlike MG-NGS, can analyze gene expression and differentiate between inactive and active genetic pathways; this is a ribonucleic acid sequencing method that affords the possibility of determining virulence and resistance to antibiotic therapy [21].

**Table 1:** Sensitivity and specificity of metagenomics next-generation sequencing from all studies included in the systematic review. mNGS: metagenomics next generation sequencing; sens: sensitivity; spec: specificity

Sensitivity and specificity studies	mNGS	Cultures	Sonication fluid cultures
Huang et al. in 2020 [21]	Sens=95.9% and spec=95.2%	Sens=79.6%, p=0.014; spec=95.2%, p=1.0	-
Street et al. in 2017 [22]	Sens=88% and spec=88%	-	Sens=68% and spec=82%
Thoendel et al. in 2018 [23]	Sens=82.9%	-	-
Ivy et al. in 2018 [24]	Sens=84% and spec=94.4%	Sens=92% and spec=100%	-
Wang et al. in 2020 [25]	Sens=95.6% and spec=94.4%	Sens=77.8% and spec=94.4%	-
Cai et al. in 2020 [26]	Sens=95.45% and spec=90.91%	Sens=72.72% and spec=77.27%	-
He et al. in 2021 [27]	Sens=95% and spec=94.7%	-	-
Yu et al. in 2023 [28]	Sens=80.6% and spec=84.6%	-	-
Fang et al. in 2020 [29]	Sens=92% and spec=91.7%	Sens=52% and spec=91.7%	-

Treatment

The treatment, both surgical and antibiotic, of PJI must be individualized. Surgical treatment includes debridement and implant retention (DAIR), one-stage implant replacement, and two-stage revision [30]. Debridement and implant retention (DAIR) involves the following: (1) excision of the fistulous tracts and the areas affected by them, (2) open arthrotomy with excision of the old scar, (3) a minimum of five biopsies are performed (pus, capsule, tissue periprosthetic, bone, joint fluid, muscle tissue, etc.), (4) extensive tissue debridement, with the removal of necrotic areas and the exclusion of interchangeable prosthetic parts, (5) pulsatile lavage under pressure with antiseptic solutions and physiological serum, (6) removal of the microbial biofilm with a wet sponge or gauze from the level of the remaining surfaces of the prosthesis, and (7) the changing of gloves after debridement, introducing new mobile components of the prosthesis [31,32].

One-stage implant replacement involves removing the old prosthesis and replacing it at the same time. Re-implantation at one time is carried out in patients with positive cultures for known bacteria that are not resistant to active biofilm antibiotics, without fistulas and without changes to bone and soft tissues; these procedures have an increased success rate, shorter periods of hospitalization, and earlier resumption of mobility, and thus, lower costs [33].

The two-stage revision is considered the gold standard in the treatment of periprosthetic infections (especially in the case of difficult-to-treat microorganisms, such as enterococci and fungi) and involves the removal of the prosthesis, with its replacement after a certain period of time (two to four weeks in the case of known infectious agents, without resistance to antibiotic therapy, and eight weeks for difficult-to-treat or unknown microorganisms) [7,33,34].

In the case of all surgical interventions, the total recommended duration of broad-spectrum antibiotic treatment is 12 weeks. Antibiotic therapy without a surgical procedure is only recommended in selected cases, such as patient refusal or cases in which the intervention is contraindicated. Rifampicin is indicated in periprosthetic infections caused by Staphylococci and Propionibacterium spp., and ciprofloxacin, in the case of Gram-negative bacteria, acting on the bacterial biofilm, with the possibility of vancomycin administration in case of allergies to cephalosporins [31].

Periprosthetic joint infections are probably the most important complication for patients undergoing total hip or total knee arthroplasty. The implications of PJIs are significant, mainly due to the impossibility of immediate and correct identification of the responsible germ. Multiple studies have shown that mNGS can be used to improve the diagnosis of periprosthetic infections, but it has some disadvantages that must be taken into account when it is used on a large scale. Most of the nucleic acids in the samples come from the host, which makes it difficult to detect the pathogen [5]. mNGS is also susceptible to bacterial contamination at some stages during its processing and interpretation of results is difficult because many contaminating organisms are also potential pathogens associated with periprosthetic infections. The use of this technique is currently limited by the high cost and complex laboratory and bioinformatics workflows required. Another study compared the results of mNGS, using samples from different types of tissue, and found that, when using sonication fluid, the sensitivity and specificity are at their highest value, respectively, 92.5% and 94.7% [28].

A study by Goswami et al. compared MT and MG and found that MT offers the unique ability to accurately detect microorganisms and ARGs on a larger scale than other existing techniques. As with all infectious diseases, accurate identification of resistant strains is essential to ensure appropriate antibiotic treatment. The results of this study showed that MT had a high, 83%, concordance rate for positive cultures [35].

Another important aspect that must be discussed is the ability of NGS to detect a potential underlying “native” joint microbial community. For a long time, it was believed that the joint represents a sterile environment devoid of microorganisms, but recent literature highlights the existence of an endemic microbiome in the joints [36,37].

Although all types of NGS can detect low levels of bacteria, contamination of collected samples remains a challenge and the risk must be reduced by all possible means. False positive results can lead to an incorrect diagnosis that can subject the patient to unnecessary surgical procedures and antibiotic treatments [38,39].

The relatively high cost of NGS, compared to standard microbiological tests, maybe a limitation of its current use, but this must be weighed against the final cost of an arthroplasty in which the germs cannot be identified and the ineffectiveness of empiric antibiotic therapy. The turnaround time of NGS can be between three and five days and mNGS can have a turnaround time of less than two days, the clinical applicability of the shorter time period being important [40,41].

## Conclusions

One of the main problems in the correct diagnosis and treatment of a periprosthetic infection is represented by the correct identification of the responsible agent. The combination of correct surgical debridement and targeted antibiotic treatment is based on the rigorous preoperative and intraoperative identification of the bacterial agent. Standardization of treatment in the absence of a correct microbiological diagnosis will lead to treatment failure and unnecessary toxicity.

mNGS is an excellent technique for identifying pathogens in periprosthetic infections, and especially for identifying the germs involved in periprosthetic infections with negative cultures, subsequently providing valuable information regarding microbial resistance to antibiotics, which will ultimately lead to an optimized therapeutic response with a shorter duration. Another advantage of mNGS compared to conventional NGS is represented by the speed of bacterial identification, which has an impact on the initiation of targeted and non-empirical antibiotic therapy and helps to avoid possible secondary toxicities. The sensitivity and specificity of mNGS are high, but this is not ideal, given the large amount of host DNA, which means that, sometimes, the bacterial DNA cannot be correctly identified. The combination of mNGS and classic microbiological techniques for bacterial identification leads to an optimization of the diagnosis of periprosthetic infections.
